# A sensitive single-enzyme assay system using the non-ribosomal peptide synthetase BpsA for measurement of L-glutamine in biological samples

**DOI:** 10.1038/srep41745

**Published:** 2017-01-31

**Authors:** Alistair S. Brown, Katherine J. Robins, David F. Ackerley

**Affiliations:** 1 School of Biological Sciences, Victoria University of Wellington, Wellington, New Zealand; 2 Centre for Biodiscovery, Victoria University of Wellington, Wellington, New Zealand

## Abstract

The ability to rapidly, economically and accurately measure L-glutamine concentrations in biological samples is important for many areas of research, medicine or industry, however there is room for improvement on existing methods. We describe here how the enzyme BpsA, a single-module non-ribosomal peptide synthetase able to convert L-glutamine into the blue pigment indigoidine, can be used to accurately measure L-glutamine in biological samples. Although indigoidine has low solubility in aqueous solutions, meaning direct measurements of indigoidine synthesis do not reliably yield linear standard curves, we demonstrate that resolubilisation of the reaction end-products in DMSO overcomes this issue and that spontaneous reduction to colourless *leuco*-indigoidine occurs too slowly to interfere with assay accuracy. Our protocol is amenable to a 96-well microtitre format and can be used to measure L-glutamine in common bacterial and mammalian culture media, urine, and deproteinated plasma. We show that active BpsA can be prepared in high yield by expressing it in the *apo*-form to avoid the toxicity of indigoidine to *Escherichia coli* host cells, then activating it to the *holo*-form in cell lysates prior to purification; and that BpsA has a lengthy shelf-life, retaining >95% activity when stored at either −20 °C or 4 °C for 24 weeks.

Glutamine is the most abundant amino acid in the human body, owing to its ability to act as the major intercellular transporter of amino-nitrogen and as a key fuel source for rapidly dividing cells, including cells of the immune system and intestinal lining^1^,^2^. Consequently, glutamine is very important in numerous aspects of medicine. Not only is it used as a common supplement for athletes or patients experiencing critical illness^1^,^3^, but abnormal levels in bodily fluids can be symptomatic of certain diseases, e.g. metabolic diseases^4^,^5^, over-training syndrome^6^,^7^ or neurodegenerative disorders^8^,^9^. Moreover, tumours can become addicted to glutamine as an energy source^10^, and this can manifest as unusually low glutamine levels in serum or saliva^11^,^12^. Glutamine is also enormously important from a research perspective as it provides a particularly useful energy source for mammalian cell culture^13^. However, excessive levels of glutamine in culture medium can inhibit the transport of other amino acids^14^ or result in the generation of toxic levels of ammonia^15^,^16^. For all of these reasons and more, it is important to be able to accurately quantify the levels of glutamine that are present in complex biological mixtures.

The “gold standard” for glutamine measurement, and method most commonly employed by hospital laboratories, is HPLC^17^. However, this method is expensive, unwieldy, and unsuited to rapid turnaround of samples. Specialized instruments have been developed to monitor the concentrations of various metabolites including glutamine in cell culture and fermentation media, but these instruments are also expensive and a typical device was recently found to be insufficiently accurate for point-of-care analysis of glutamine levels in patient plasma samples^17^.

In contrast, enzymatic methods offer promise for rapid, accurate and cost-effective quantification of glutamine in diverse biological samples. The primary method that has been used to achieve this, employed in a wide range of commercially available kits, is a two-step enzymatic conversion of glutamine to glutamate by glutaminase followed by a further deamination of the glutamate to α-ketoglutarate by glutamate dehydrogenase^18^. As the second step is accompanied by a proportional reduction of NAD^+^ to NADH, the reaction can be monitored spectrophotometrically. However, not only does this method involve multiple reagents and time-consuming reaction steps, it also requires that the baseline of glutamate in the sample be established separately, so that this can be subtracted from the combined (glutamine + glutamate) measurement. This extra step introduces additional error and potential for cross-sample contamination.

We present here an alternative means for assaying glutamine, based on a single enzymatic conversion of two molecules of L-glutamine into the directly detectable blue pigment indigoidine (Fig. 1), by the single-module non-ribosomal peptide synthetase (NRPS) BpsA (Blue pigment synthetase A). Indigoidine has both antioxidant and antimicrobial properties^19^,^20^, and BpsA was originally discovered in *Streptomyces lavendulae* by Takahashi *et al*.^21^ Subsequent applications of this enzyme have included as an *in vivo* reporter^22^,^23^,^24^, as a tool for discovery^25^ and analysis^26^ of phosphopantetheinyl transferase (PPTase) enzymes, and as a model for NRPS domain recombination experiments^27^,^28^. However, no one has previously adapted BpsA for measurement of glutamine, most likely because the unusual and poorly understood solution chemistry of indigoidine results in unusual absorbance profiles (Fig. 1C) from which it is not obvious how a linear standard curve might be generated. We demonstrate here that a final solubilisation step can effectively resolve this issue.

## Results

### Expression and purification of functional *holo*-BpsA

**Figure 1:**
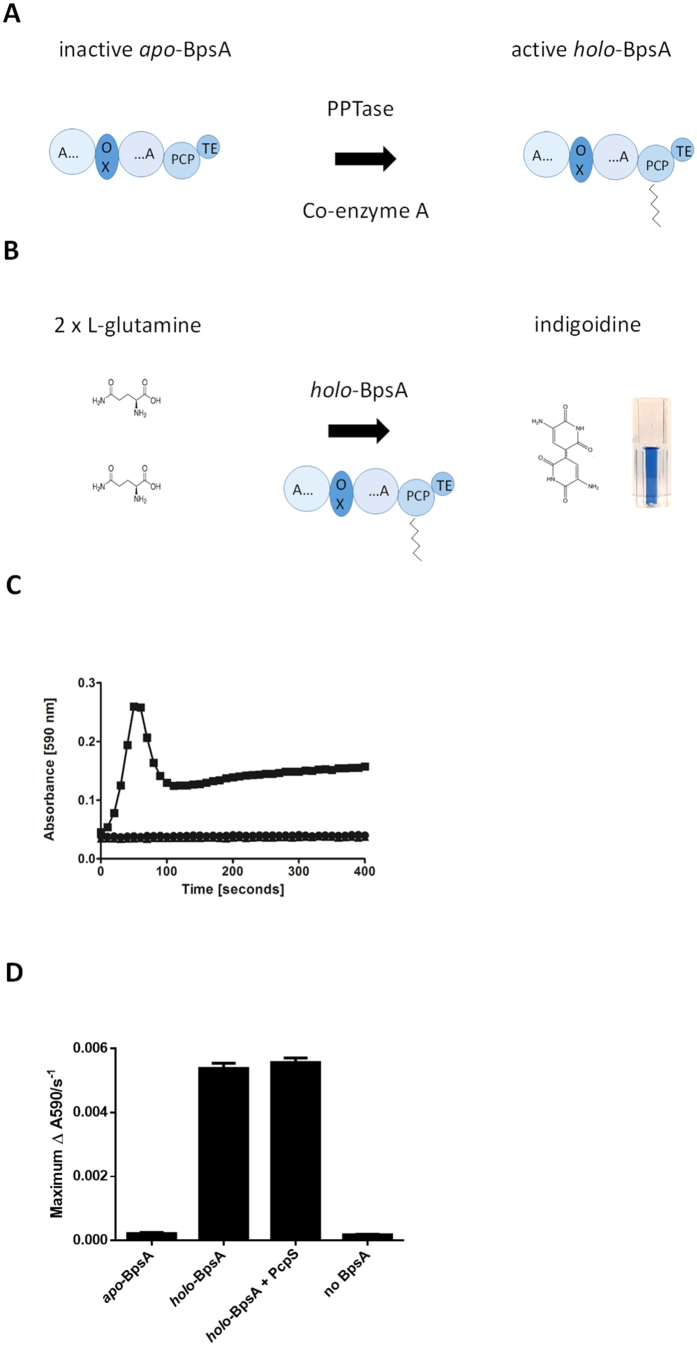
Production of indigoidine by the single-module NRPS BpsA. (**A**) Schematic diagram showing the activation of BpsA via attachment of a 4′-phosphopantetheine (PPT) prosthetic group derived from coenzyme A, mediated by a PPTase enzyme. BpsA consists of an adenylation (A) domain interrupted by an oxidation (Ox) domain, a peptidyl carrier protein (PCP) domain and a thioester (TE) domain. (**B**) Schematic diagram showing two molecules of L-glutamine being converted by *holo*-BpsA into the easily detectable blue pigment indigoidine. (**C**) *Holo*-BpsA is able to rapidly synthesise indigoidine from two molecules of L-glutamine, whereas *apo*-BpsA is unable to synthesise indigoidine. Three reaction mixes containing either 1 μM *apo*-BpsA (●), *holo*-BpsA (■) or no BpsA (▲), in 50 mM Tris-Cl pH 8.5, 20 μM MgCl_2_ and 6 μM ATP, were set up in a 96-well plate. The reactions were initiated by the addition of 1000 μM L-glutamine (final concentration, bringing the total reaction volume to100 μL). A_590_ values were monitored every 10 s. The graph shows the characteristic increase in absorbance as indigoidine is synthesised by *holo*-BpsA. In contrast, *apo*-BpsA is unable to synthesise indigoidine so there is no increase in A_590_. Data are the mean values from two independent experiments, each comprising three technical replicates, and error bars indicate standard error of the mean. (**D**) Incubation of *holo*-BpsA with purified PcpS and coenzyme A does not further increase the rate of indigoidine synthesis. Four otherwise identical reaction mixes were set up in triplicate containing either 2 μM *apo*-BpsA, 2 μM purified *holo*-BpsA, 2 μM purified *holo*-BpsA that was further incubated with 0.25 μM of purified PcpS, or ddH_2_O in place of BpsA (i.e., a no-BpsA control). Following a 30 min incubation, indigoidine synthesis was initiated by the addition of a second reaction mix containing ATP and L-glutamine. Further incubation with purified PcpS made little difference to the maximum rate of indigoidine synthesis, indicating that the majority of BpsA had already been successfully converted into the *holo* form during the mixed cell lysate incubation. Data are the mean values of three replicates and error bars indicate standard error of the mean.

Like all NRPS enzymes, in order for BpsA to synthesise its product (i.e., indigoidine) it first needs to be converted from an inactive *apo* form to an active *holo* form^21^. This conversion is achieved by the attachment of a 4′-phosphopantetheine (PPT) moiety derived from coenzyme A to the peptidyl carrier protein domain of BpsA, catalysed by a 4′-phosphopantetheinyl transferase (PPTase) enzyme^29^ (Fig. 1A). A variety of PPTases from different bacterial species have been shown to be able to activate BpsA, with one of the more effective PPTases being PcpS from *Pseudomonas aeruginosa*^26^. However, PPT attachment is difficult to achieve *in vivo* prior to BpsA purification, as the mild anti-bacterial properties of indigoidine^19^ inhibit the growth of *E. coli* cells that are producing *holo*-BpsA^26^. To avoid production of *holo*-BpsA *in vivo*, BpsA was expressed as a His6-tagged protein in a strain of *E. coli* BL21(DE3) that had the endogenous non-essential PPTase gene *entD* knocked out as previously described^25^. Prior to purification, conversion of His6-tagged *apo*-BpsA to the *holo* form was achieved by mixing the soluble fraction of cell lysate with lysate from *E. coli* BL21 cells that were over-expressing non-His6-tagged PcpS, together with excess coenzyme A as a source of PPT. *Holo*-BpsA was then purified via nickel affinity chromatography, and activity was confirmed by monitoring indigoidine production in the presence of L-glutamine and ATP (Fig. 1C). Further incubation of this enzyme with His6-tagged PcpS that had been purified separately did not lead to an increase in the rate of indigoidine synthesis (Fig. 1D). Although 100% conversion to the *holo* form is not essential for an assay system where all samples are tested using the same enzyme preparation, this observation nevertheless suggests that near-complete conversion of BpsA to the *holo* form had been achieved during the mixed lysate incubation step.

### Development of an assay to quantify indigoidine production

**Figure 2:**
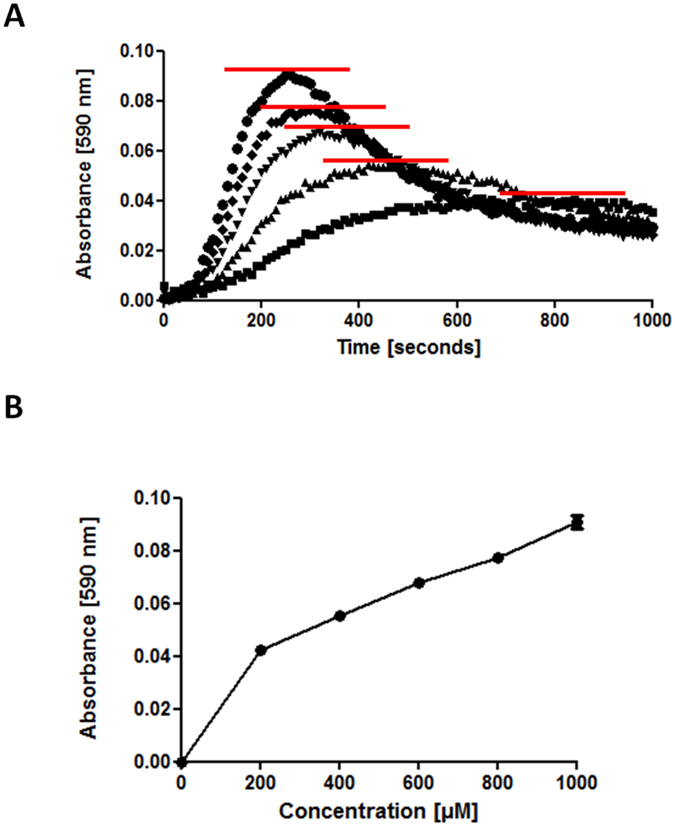
Direct monitoring of indigoidine synthesis does not yield a linear standard curve. (**A**) A master mix containing 2 μM *holo*-BpsA, 50 mM Tris-Cl pH 8.5, 20 mM MgCl_2_, 5 mM ATP and ddH_2_O a final volume of 90 μl was added to individual wells of a 96 well plate. To initiate the reaction, 10 μL of L-glutamine stock solutions were added to the following concentrations 1000 μM (●), 800 μM (♦), 600 μM (▼), 400 μM (▲), 200 μM (■) or 0 μM L-glutamine, A_590_ values were recorded for each well every 20 s, and the 0 μM background (a flat-line) was subtracted from each set of data. Each data point pictured is the average of three technical replicates. The red lines mark the peak absorbance value observed for each L-glutamine concentration. (**B**) A standard curve was generated from the normalised peak absorbance values recorded for each L-glutamine concentration. Data are the mean values of three technical replicates, and error bars indicate standard error of the mean.

Indigoidine synthesis in an aqueous solution does not yield an asymptotic curve of absorbance over time. Instead of being a smooth curve that levels off as a maximum A_590_ value is approached, a typical indigoidine synthesis curve rapidly reaches a first maximum, after which the A_590_ declines equally rapidly before slowly increasing once again (Fig. 1C). Although greater starting concentrations of L-glutamine in a test solution result in higher initial A_590_ maxima (Fig. 2A), we found that plotting these maxima from samples containing a range of known L-glutamine concentrations yielded a curved rather than linear series of points (Fig. 2B). We did find that plotting the initial maximal rates of indigoidine synthesis (i.e., the steepest slope of each individual curve) was sometimes capable of yielding a linear set of values suitable for generation of a standard curve (e.g., Supplementary Figure S1), however this outcome was not reproducible at different *holo*-BpsA concentrations, and we considered that the kinetic nature of the assay was more prone to variability than an end-point assay would be.

**Figure 3:**
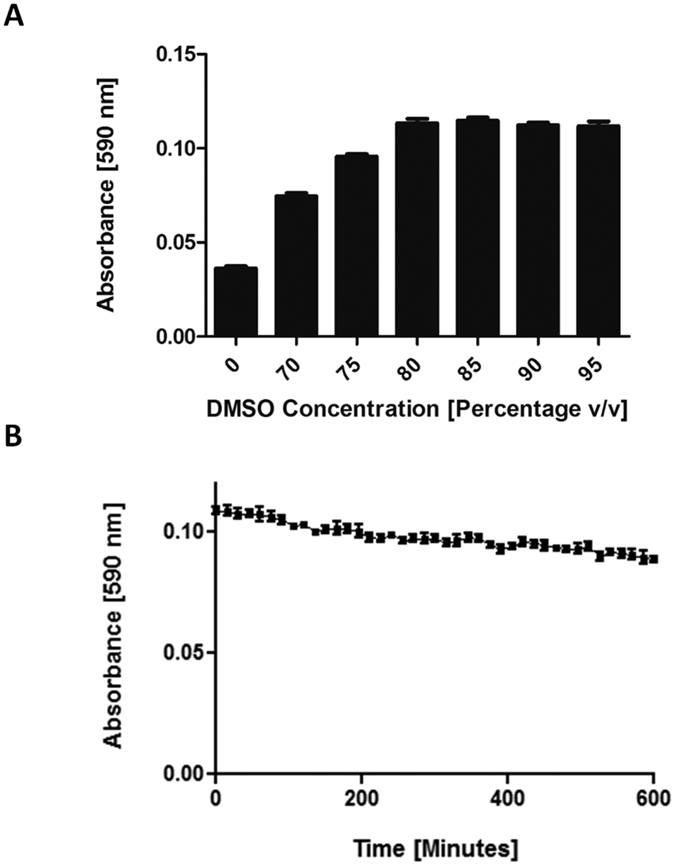
Resolubilisation of indigoidine in the inorganic solvent DMSO enables a linear standard curve to be generated. (**A**) Replicate reaction mixes containing 50 mM Tris-Cl pH 8.5, 20 mM MgCl_2,_ 12 mM ATP, 3 μM *holo*-BpsA, 5 mM L-glutamine and ddH_2_O to a total volume of 40 μL were incubated for 1 h at 25 °C. Addition of DMSO to final concentrations of 80% or higher were found to maximise the solubility of indigoidine present in an aqueous solution, enabling more accurate quantification via measurement of absorbance at 590 nm. (**B**) The A_590_ of a fully solubilised 200 μL solution of indigoidine in 95% DMSO (■) was found to diminish only slightly over a 10 h period, consistent with indigoidine undergoing a gradual conversion into the colourless leuco isoform. Data are the mean values from two independent experiments, each comprising three technical replicates, and error bars indicate standard error of the mean.

It is known that indigoidine can spontaneously be reduced to a colourless *leuco* isoform^21^,^22^, and we considered that this conversion might be one contributing factor to the characteristic absorbance profile of indigoidine production over time. However, visual examination of the wells after prolonged synthesis of indigoidine indicated a dominant factor was more likely that indigoidine was dropping out of solution, evidenced by formation of a slight blue precipitate. It has previously been shown that indigoidine is soluble in a limited range of organic solvents (including DMSO, THF, NMP, and DMF)^30^. DMSO was selected for use here, owing to its minimal toxicity and general availability in the laboratory setting. To test whether indigoidine could be effectively resolublised in DMSO under our assay conditions, we first synthesised indigoidine in 40 μL replicates of reaction mix. 760 μL of various ratios of ddH_2_O and DMSO were then added to individual replicates to bring the total volume up to 800 μL, after which resolubilisation was attempted by shaking at 2,000 rev/min for 20 min. We found that indigoidine became fully soluble at final concentrations of 80% DMSO (v/v) and above (Fig. 3A). Resolubilisation of indigoidine also allowed us to establish that spontaneous conversion to a colourless *leuco* form was not substantially impairing our ability to accurately estimate L-glutamine concentrations within the timeframe of our assay. To test this, the absorbance of 200 μL of solubilised indigoidine in 95% DMSO, prepared as per Fig. 3A, was monitored at 590 nm for 10 h at 25 °C (Fig. 3B). While the absorbance readings did decrease slowly over time, we concluded that the rate of decrease was insufficient to interfere with accurate measurement of L-glutamine within the timeframe of our assay.

Based on these observations we reasoned that it should be possible to both miniaturise the assay into a 96 well plate format and generate robust linear standard curves, by first converting all L-glutamine in a sample solution to indigoidine in a 96 well microtitre plate, and then stopping the reaction and re-solubilising the indigoidine via the addition of DMSO to a final concentration of approximately 80% (v/v), followed by measurement of the final absorbance at 590 nm.

### Miniaturisation of BpsA incubation and indigoidine solubilisation protocol

To accurately quantify the amount of L-glutamine present in a sample using BpsA it is necessary to catalyse the complete conversion to indigoidine, followed by the complete solubilisation of the indigoidine formed. It was therefore essential to employ a reaction volume that would still allow DMSO to be added to a final concentration of at least 80% (v/v) prior to A_590_ measurement. We found that using 30 μl of reaction mix and 10 μL of sample was sufficient to reliably achieve this in a standard 96 well flat bottomed microplate having a well volume of 360 μL. After the conversion of L-glutamine to indigoidine was completed, 200 μL of anhydrous DMSO was added to the original 40 μL reaction volume, resulting in a final concentration of 83% DMSO.

**Figure 4:**
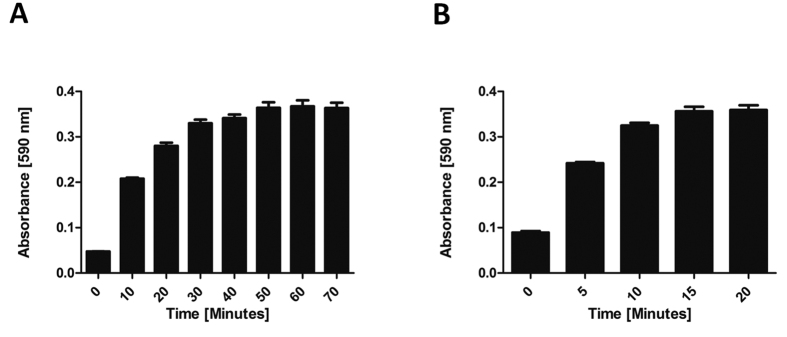
(**A**) For a reaction mix comprising 1000 μM L-glutamine, 50 mM Tris-Cl pH 8.5, 10 mM MgCl_2_, 5 mM ATP and 3 μM *holo*-BpsA, increasing the reaction time to 50 min resulted in higher final A_590_ values, indicating greater conversion of L-glutamine to indigoidine. Beyond 50 min, no additional indigoidine production was measurable. Data are the mean values from two independent experiments, each comprising three technical replicates and error bars indicate standard error of the mean. (**B**) Increasing the incubation time to 15 min post-addition of 83% (v/v) DMSO was found to increase the A_590_ signal generated due to solubilisation of indigoidine. After 15 min no further indigoidine solubilisation was observed. Data are the mean values from two independent experiments, each comprising three technical replicates, and error bars indicate standard error of the mean.

We next sought to identify a suitable reaction time to allow all of the L-glutamine in the reaction mix to be converted to indigoidine prior to addition of DMSO. For this, replicate 10 μL samples of 1 mM L-glutamine in ddH_2_O were individually added to 30 μL aliquots of reaction mix (comprising 50 mM Tris-Cl pH 8.5, 10 mM MgCl_2,_ 5 mM ATP, 3 μM *holo*-BpsA). and incubated at 25 °C with shaking at 200 rev/min. At 10 min intervals, groups of three replicates were stopped by the addition of 200 μL DMSO. It was found that under these conditions complete conversion of L-glutamine to indigoidine had occurred within 50 min (Fig. 4A).

It was also important to ensure complete solubilisation of the indigoidine, to enable accurate A_590_ readings. To identify a suitable incubation time for resolubilisation of indigoidine in a final concentration of 83% DMSO (v/v), replicate reaction mixes as detailed above were incubated at 25 °C and 2,000 rev/min, with A_590_ readings taken every 5 min. After 15 min the indigoidine appeared to be completely solubilised (Fig. 4B).

### Generation of a standard curve using the optimised protocol

**Figure 5:**
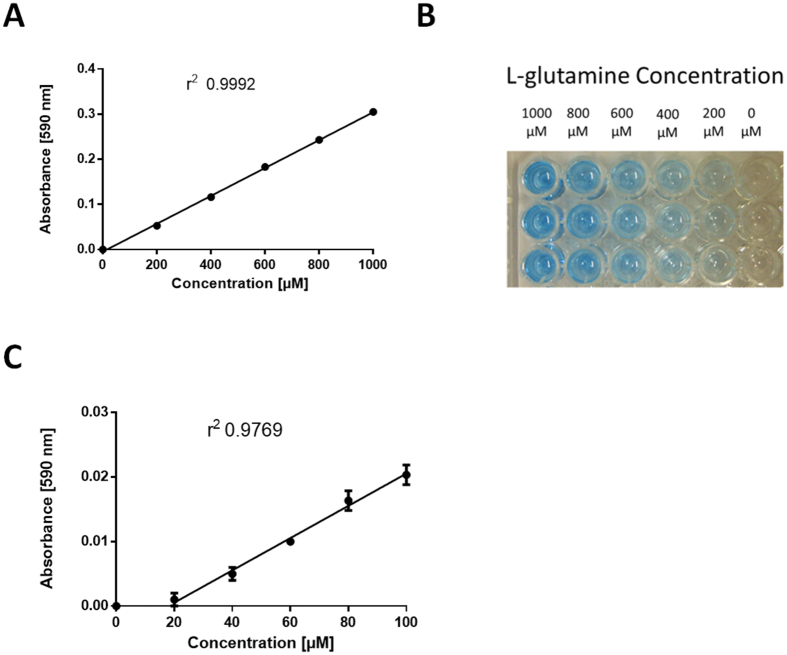
(**A**) A linear standard curve was generated by incubating 10 μL L-glutamine standards at a range of concentrations (0 to 1000 μM) with 30 μL of reaction mix (50 mM Tris-Cl pH 8.5, 10 mM MgCl_2,_ 5 mM ATP, 3 μM *holo*-BpsA in ddH_2_O) for 1 h at 25 °C. This was followed by resolubilisation in 200 μL DMSO and a further incubation with shaking at 2,000 rev/min for 20 min at 25 °C. Data are the means of three replicates and error bars indicate standard error of the mean. The data was normalised to zero for the 0 μM L-glutamine standard, and the r^2^ value was calculated using Graphpad Prism. (**B**) An image of a standard curve established as for panel A, showing the pigment intensity proportionate to the starting levels of L-glutamine present in each sample. Three replicates are shown. (**C**) A linear standard curve established as per panel A, only using 10 μL of L-glutamine standards with a concentration range of 0–100 μM. No signal was detectable below 20 μM L-glutamine.

Using these optimised reaction conditions (1 h reaction incubation, with a 30 μL reaction mix comprising 50 mM Tris-Cl pH 8.5, 10 mM MgCl_2,_ 5 mM ATP, 3 μM *holo*-BpsA, followed by resolubilisation in 200 μL DMSO and a further incubation with shaking at 2,000 rev/min for 20 min at 25 °C), we sought to test the accuracy of the assay in measuring a range of L-glutamine concentrations in 10 μL samples. Using standards of a 10 mM stock solution of L-glutamine diluted to give a range of 0–1000 μM in ddH_2_O, we showed that we could now generate highly reproducible linear standard curves having excellent r^2^ values (e.g., 0.9992; Fig. 5A,B). Empirical testing revealed that the reaction was linear to approximately 1500 μM L-glutamine, provided the incubation times for indigoidine formation and re-solubilisation were also increased (not shown). A similar curve using 10 μL standards of L-glutamine with a lower concentration range (0–100 μM in ddH_2_O) had a lower r^2^ value (0.9769; Fig. 5C), indicating greater variability within this range, albeit still a high level of accuracy. No difference in signal was observed between the 0 μM and 20 μM standards, indicating that 20 μM L-glutamine is the detection limit of the assay using these parameters.

In parallel, we considered whether conversion of indigoidine to its *leuco* form using a protocol previously established by Müller *et al*.^22^ might provide an alternative means of accurately measuring L-glutamine concentrations. To test this, we first generated an indigoidine standard curve as for Fig. 5A, using 10 μL samples of L-glutamine standards from 0–1000 μM (Supplementary Figure S2A). We then added 2.3 μL of the reducing agent sodium dithionite to each well, resulting in a complete conversion to the colourless *leuco* form. Subsequently, a standard curve was generated by monitoring the fluorescence of each well (ex 415 nm/em 520 nm; Supplementary Figure S2B). The r^2^ value for the *leuco* fluorescence standard curve (0.4597) was far lower than the colorimetric indigoidine standard curve (0.9938), indicating that the former was a less accurate method for quantifying L-glutamine.

We also considered that it might be advantageous for certain applications to decrease the incubation time required for conversion of L-glutamine to indigoidine. We reasoned that the simplest way to achieve this would be to increase the concentration of enzyme in the assay. When we increased the concentration of *holo*-BpsA 5-fold from the optimised reaction conditions described above (i.e., to 15 μM) we observed that near-complete conversion of L-glutamine to indigoidine was achieved within 15 min of incubation (Supplementary Figure S3).

### Assay performance in common laboratory growth media

**Figure 6:**
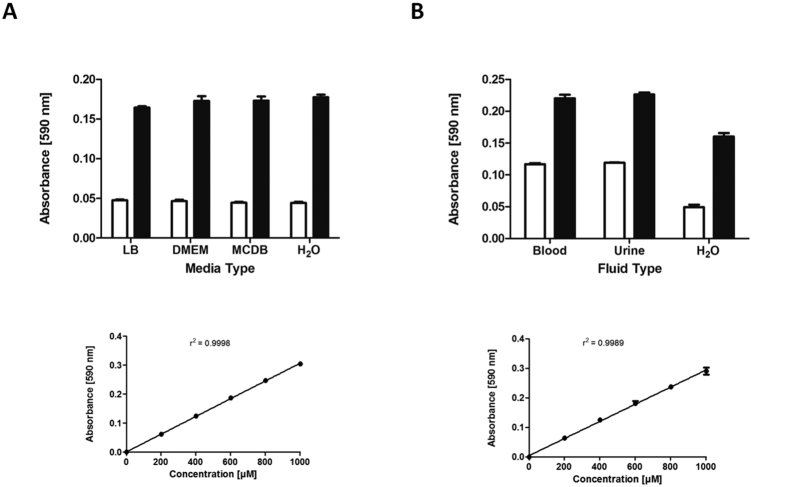
(**A**) Spiked samples of L-glutamine were measurable in a range of common growth media. 30 μL of reaction mix (50 mM Tris-Cl pH 8.5, 10 mM MgCl_2,_ 5 mM ATP, 3 μM *holo*-BpsA in ddH_2_O) were added to each well. Test samples consisting of either 10 μL unamended media (white bars), or 10 μL media to which had been added 400 μM L-glutamine (black bars), were added to each well. The reactions were then incubated for 1 h at 25 °C to fully convert the L-glutamine into indigoidine, after which samples were resolublised by addition of 200 μL DMSO incubated at 2,000 rpm for 20 min. For the standard curve and the derived data presented in Table 1, data was normalised to zero for the 0 μM L-glutamine standard, and the r^2^ value was calculated using Graphpad Prism. All data are the means of three replicates and error bars indicate standard error of the mean. (**B**) Biological fluids were assayed for L-glutamine, using spiked samples in the same manner as panel A. Test samples consisted of either urine or de-proteinated plasma, with ddH_2_O added at a 1:1 ratio. For the standard curve and the derived data presented in Table 1, data was normalised to zero for the 0 μM L-glutamine standard, and the r^2^ value was calculated using Graphpad Prism. All data are the means of three replicates and error bars indicate standard error of the mean.

Possible applications of a glutamine quantification assay include measuring levels of the essential but unstable additive L-glutamine in mammalian cell culture media, and measuring levels of L-glutamine yield from an industrial bacterial producer strain in bacterial culture media. To test whether our BpsA assay could accurately quantify L-glutamine levels in diverse culture media, samples of Lysogeny Broth (LB) and two mammalian cell culture media, DMEM (Dulbecco’s Modified Eagle Medium) and MCDB (Molecular, Cellular, and Developmental Biology) medium were each spiked with 400 μM L-glutamine and compared against non-spiked controls. The values for the non-spiked media did not show any intrinsic variation when compared to water, indicating that there was no L-glutamine present in the unamended media and that the colour of each medium did not fundamentally interfere with the detection of indigoidine (Fig. 6A). Importantly, the levels of indigoidine measured in each L-glutamine supplemented medium were reproducible and accorded with the 400 μM spiking level (Table 1).

### Assay performance in clinically relevant biological fluids

We next sought to determine whether the BpsA assay could also be used to measure L-glutamine concentrations in biological fluids such as blood and urine. Although urine is known to vary in pH depending on factors such as fluid and food intake, it has previously been shown that *holo-*BpsA is functional across a wide pH range^26^, offering promise that the assay would be effective with urine samples (in contrast with the standard metabolite profiling technique of Gas Chromatography Mass Spectrometry, which is unreliable for quantification of L-glutamine in urine^31^). To test this, a fresh urine sample was first diluted 1:1 (v/v) in ddH_2_O (due to the reported concentrations of L-glutamine in blood and urine being above 500 μM, which might cause the concentration of a spiked sample to be above 1 mM^32^). The diluted sample was then assayed in parallel with a sample that had been spiked with 400 μM additional L-glutamine. The resulting A_590_ values showed a consistent difference between spiked and un-spiked samples (Fig. 6B, Table 1) indicating that the *holo-*BpsA enzyme was effective at measuring L-glutamine concentrations in urine.

In contrast, initial attempts to measure the concentration of L-glutamine in plasma failed. The addition of DMSO to the reaction mix containing 10 μL of plasma sample caused the mix to become cloudy and prevented accurate A_590_ readings from being obtained. We hypothesised this was due to the DMSO reacting with the proteins and cell debris present in the plasma, on the basis of empirical tests that revealed indigoidine could readily be generated and measured in commercially sourced adult bovine serum. To remove these confounding constituents, we first pre-treated the plasma by passing it through a column with 3 kDa retention cut-off. Following this step, we found we could now accurately measure the L-glutamine present in the serum. Samples consisting of a 1:1 (v/v) mixture of ddH_2_O and deproteinated blood were assayed and directly compared to replicate samples that had been spiked with an additional 400 μM of L-glutamine. The resulting absorbance values clearly show an increase in absorbance with the spiked blood sample, corresponding to the predicted 400 μM increase in L-glutamine content (Fig. 6B, Table 1).

It is possible that the sample background might exert subtle effects on assay variability, as although our measurements of L-glutamine in the different culture media and biological fluids were consistent with the spiked levels, the errors associated with these measurements were typically higher than for the L-glutamine standards in ddH_2_O (e.g., Fig. 5A). Nevertheless, our results show that the BpsA assay is generally robust for use in these different applications.

### Evaluation of the shelf life of BpsA

**Figure 7:**
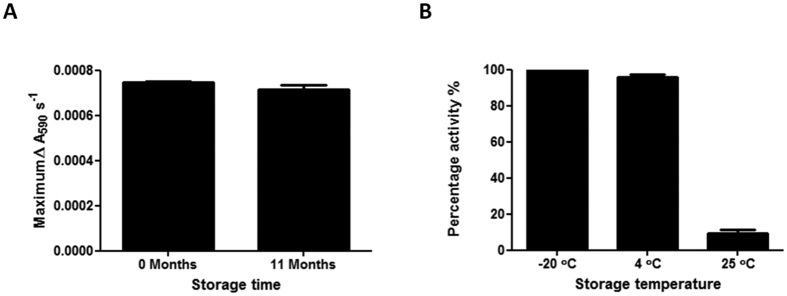
(**A**) *Holo-*BpsA was assayed for activity (maximal reaction velocity) before and after storage at −20 °C for 11 months. In each case a master mix containing 2 μM *holo*-BpsA, 50 mM Tris-Cl pH 8.5, 20 mM MgCl_2_, 5 mM ATP and ddH_2_O to a final volume of 90 μL was dispensed into individual wells of a 96 well plate. The reaction was initiated by the addition of 10 μL 1000 μM L-glutamine in ddH_2_O. The 96 well plate was shaken at 1000 rev/min for 10 s and the A_590_ values were recorded every 10 s for 1 h. The maximal velocity of the reaction was calculated by finding the maximum slope value as previously described^26^. Data are the means of three replicates and error bars indicate standard error of the mean. (**B**) BpsA was assayed for activity (maximal reaction velocity) before and after storage at either −20 °C, 4 °C or 25 °C for 24 weeks. To convert the *apo-*BpsA to *holo*-BpsA prior to activity assays the following reaction mix was used: 2 μM BpsA, 12.5 μM Co-enzyme A, 0.1 μM Sfp, 5 mM MgCl_2_, 50 mM Tris pH 7.8 and ddH_2_O to a total volume of 25 μL per reaction. Reactions were incubated at 30 °C with shaking at 200 rev/min for 30 min to ensure complete conversion to *holo-*BpsA. Next, 25 μL of the *holo*-BpsA mix was dispensed into individual wells of a 96 well plate, each containing 50 μL of 50 mM Tris-Cl pH 7.8 and 5 mM MgCl_2_ in ddH_2_O. Indigoidine synthesis was initiated by the addition of 25 μL of 5 mM ATP and 2 mM L-glutamine in ddH_2_O (concentrations are per final 100 μL reaction volume). The 96 well plate was then incubated at 25 °C with shaking at 1000 rev/min for 10 s and the A_590_ values were recorded every 20 s for 1 h. The velocity of the reaction was calculated by finding the maximum slope value as above, and percentage activity was calculated for each sample relative to the pre-storage level of activity. The graph bars are the mean of three replicates and the error bars indicate the standard error of the mean.

For BpsA to be broadly useful it is necessary that the enzyme retain stability and activity for extended periods of time, so that it need not be purified anew prior to each assay. To evaluate the shelf life of the enzyme, a preparation of *holo*-BpsA was generated and its maximal rate of indigoidine synthesis measured as (7.48 ± 0.03) × 10^−4^ ΔA_590_ s^−1^ in triplicate assays using a master mix that included 1000 μM L-glutamine. The *holo-*BpsA was then stored at −20 °C in storage buffer (50 mM sodium phosphate buffer pH 7.8 containing 40% (v/v) glycerol). When this activity assay was repeated 11 months later the mean maximal rate of indigoidine synthesis was measured as (7.18 ± 0.17) × 10^−4^ ΔA_590_ s^−1^, indicating that BpsA retains >95% activity during long term storage at −20 °C (Fig. 7A).

We considered that it would also be beneficial if the enzyme were at least moderately stable at 4 °C and 25 °C, so that it does not lose activity during routine handling. To test this, BpsA was prepared and stored (in storage buffer, as above) in the *apo*-form and was converted into the *holo*-form immediately prior to kinetic measurements. Following storage for 24 weeks at 4 °C it was found that nearly full activity was retained (95.7 ± 1.8%) compared to the sample stored at −20 °C (Fig. 7B). In contrast, the sample stored at 25 °C had retained only 9.1 ± 1.9% of the starting level of activity after 24 weeks.

Collectively, these data indicate that BpsA is stable for at least 11 months and its activity is unlikely to be significantly impaired by routine handling. The enzyme can be stored effectively in either a freezer or refrigerator, however the optimal storage temperature is −20 °C.

## Discussion

BpsA is well suited for use in quantitative *in vitro* assays. Compared to other NRPSs, it is a small and simple enzyme. Following its conversion to the *holo* form by a PPTase partner, it is fully autonomous in its ability to generate a pigmented product from L-glutamine and ATP. Also unusual for an NRPS enzyme, it is easily purified in a soluble form via common purification methods such as nickel affinity chromatography. Other important advantages of BpsA in this context include its tolerance to a relatively wide pH range, its sensitivity and ability to function in diverse media compositions, and its lengthy shelf life during refrigerated storage. One minor limitation however is that due to the mild antimicrobial properties of indigoidine it is difficult to express BpsA in *E. coli* in its active *holo* form. To circumvent this, we instead expressed BpsA in the inactive *apo-*form and added the PPT prosthetic group during the purification process. By mixing the lysate of an *E. coli* culture expressing His_6_ tagged *apo-*BpsA with that from an *E. coli* culture expressing non-tagged PcpS, we found we could quickly generate large amounts of *holo*-BpsA in a cost-effective manner. Mixing crude lysates also allowed us to take advantage of the coenzyme A naturally present in those lysates, likely reducing the amount of exogenous coenzyme A required to be added during the activation process.

Initial attempts to develop BpsA as a biosensor were frustrated by the insolubility of indigoidine in aqueous media, the characteristic rapid rise and then drop in A_590_ values during synthesis making it difficult to obtain accurate measurements. While there was some correspondence between the glutamine concentration present in a starting sample and the maximal rate of indigoidine synthesis or the initial maximum A_590_ value reached, it was not possible to consistently generate a reliable linear standard curve from either of these parameters. The addition of a DMSO solubilisation step not only resolved these issues, but also facilitated miniaturisation of the assay, and meant a single end-point reading could be taken rather than requiring continuous monitoring of the reaction. It is also possible that the mild oxidant properties of DMSO had the added effect of inhibiting conversion to the colourless *leuco* form of indigoidine, enhancing the robustness of our assay.

One exceptional scenario was encountered when attempting to measure the glutamine concentration in blood, where the addition of DMSO caused the solution to become opaque, interfering with A_590_ measurement in a standard platereader. To test whether the opaque effect was being caused by DMSO-mediated aggregation of proteins and other cell debris present in the blood plasma, we removed the red blood cells by centrifugation then purified the supernatant by passing it through a 3 kDa cut-off column. These processing steps caused the plasma to turn from a pale orange/pink colour to a clear fluid, which was then amenable to BpsA-mediated glutamine quantification.

Current commercially available enzymatic based methods for the measurement of L-glutamine are generally based on a combination of the enzymes glutaminase and glutamate dehydrogenase, and require two consecutive deamination reactions to take place. Not only does this method add an additional reaction step, it also importantly means that if a sample might contain both glutamine and glutamate then both “before” and “after” measurements need to be taken. The additional conversion and measurement steps add complexity, additional reagents and longer processing times. For example, the GLN1 kit marketed by Sigma Aldrich requires eight different reagents and multiple incubation steps, and moreover is difficult to miniaturise and rather expensive. Our method only requires one enzyme, a buffering solution containing MgCl_2_, Tris-Cl and ATP, and DMSO as a stop solution. The requirement for only a single reaction to convert L-glutamine into indigoidine, followed by a simple re-solubilisation step, also improves assay speed. Moreover, should greater assay speed be required, we have shown that the incubation times reported here can be greatly reduced by increasing the amount of *holo*-BpsA enzyme added to each reaction. Finally, the high-level stability of the enzyme is amenable to storage in a ‘kit’ format, and the linear range of our assay (20–1500 μM) is well suited for direct measurement of L-glutamine concentrations in blood^5^,^33^ and urine^31^ (however some cell culture media might have to be diluted 2–4 fold prior to measurement to enable their initial L-glutamine concentrations to fall within this range^34^).

In addition to the L-glutamine assay we describe here, BpsA has previously been used as a biosensor in several applications including detection of the inhibition of PPTases as novel antibiotic targets^26^, identification of novel bioactive gene clusters from eDNA libraries^25^, and in both bacterial and mammalian cells to act as a robust reporter system^22^,^27^,^28^. We propose that it might also have utility as a reporter for strains of bacteria that are used to produce commercial quantities of L-glutamine in an industrial setting, possibly even enabling directed strain improvement experiments to increase output. Our discovery that DMSO may be added to increase the accuracy of the measurements taken may improve assay accuracy and sensitivity in many of these other applications.

## Methods

### Materials and reagents

Chemicals, reagents and media used in this study were obtained from Sigma-Aldrich (St Louis, MO, USA) or Thermo Fisher Scientific (Waltham, MA, USA), unless otherwise stated. L-glutamine was purchased from Sigma-Aldrich (St Louis, MO, USA) Restriction enzymes were purchased from New England Biolabs (NEB; Ipswich, MA, USA). IPTG (isopropyl β-D-thiogalactoside) was supplied by Bioline (London, UK). T4 DNA ligase was supplied by Thermo Fisher Scientific.T4 polymerase was supplied by Thermo Fisher Scientific.

### Plasmid Construction

The BpsA expression plasmid pCDFDUET1::*bpsA* was as previously described^25^. NOHISPET, a modified version of pET28a(+) with the N-terminal Histidine tag removed was constructed from this by digesting pET28a(+) with NcoI and SalI. The digested plasmid was then blunt ended using T4 polymerase and circularised using T4 DNA ligase. The PPTase gene *pcpS* was amplified from *P. aeruginosa* PAO1 genomic DNA using the primers CCCCAAAAGCTTATGCGCGCCATGAAC and CCCCCTCGAGTCAGGCGCCGACCGC (restriction sites are underlined), and the amplification product was ligated between the HindIII and XhoI restriction sites of NOHISPET.

### Protein expression

All protein expression was conducted using the ∆ *entD* mutant of *E. coli* BL21(DE3) (as previously described^25^) as a host strain. Cultures were grown in lysogeny broth (LB) amended with plasmid appropriate antibiotics (kanamycin at 50 μg/ml and spectinomycin 100 μg/ml). 400 ml expression cultures of *E. coli* expressing either PcpS or *apo-*BpsA were inoculated to an OD600 of 0.1 from an overnight culture and incubated (37 °C, 200 rev/min) until an OD600 of 0.6–0.7 was reached. The cultures were then incubated on ice for 30 min with occasional swirling to ensure even cooling. Protein expression was induced by the addition of IPTG to a final concentration of 0.5 mM. The cultures were incubated for 24 h (18 °C, 200 rev/min) before harvesting by centrifugation (2700 *g*, 20 min, 4 °C). Cell pellets were then stored at −80 °C for at least 12 h. Typically 20–40 mg of *apo-*BpsA and 2–5 mg of PcpS were purified per litre of *E. coli* culture.

### Conversion from *apo*-BpsA to *holo*-BpsA

Cell pellets were re-suspended in a modified binding buffer (5 mM imidazole, 0.5 M NaCl, 12.5% (v/v) glycerol and 20 mM Tris-Cl pH 7.8). The resuspended pellets of cells expressing PcpS and *apo-*BpsA were mixed together and mechanically lysed using an ice cold French Press Cell. The soluble fraction was collected by centrifugation (26,000 *g*, 20 min, 4 °C), supplemented with 100 μL of 50 mM coenzyme A (CoA) and incubated for 2 h (25 °C, 200 rev/min) to facilitate the PcpS mediated attachment of 4′-phosphopantetheine arm derived from CoA to BpsA. The BpsA, PcpS and CoA mixture was then centrifuged (26,000 *g*, 20 min, 4 °C) to remove precipitated PcpS, prior to purification of the *holo*-BpsA by Ni-NTA chromatography.

### Purification of *holo*-BpsA and PPTases

Standard Ni-NTA chromatography reagents from Novagen were used according to the manufacturer’s instructions with the following protocol modification for BpsA: 12.5% (v/v) glycerol was added to each of the bind and elution buffers. After the binding step the column was then washed by the addition of 50 ml of bind buffer supplemented with 12.5% (v/v) glycerol. The wash buffer step was omitted. 8 ml of eluted fraction was collected and the buffer was exchanged for 50 mM sodium phosphate buffer and 12.5% (v/v) glycerol pH 7.8 using a 100 kDa cut off column. The buffer composition was adjusted to 40% (v/v) glycerol and aliquots were stored at −20 °C. For the purification of PPTases the standard manufacturer’s protocol was followed except the bind and elute buffers were supplemented with 25% (v/v) glycerol. The eluted protein was desalted using a desalting column and a desalting buffer containing (50 mM Tris-Cl buffer pH 7.8, 12.5% (v/v) glycerol). The buffer composition was adjusted to 40% glycerol and aliquots were stored at −80 °C.

### Further incubation of purified *holo-*BpsA with purified PcpS

Purified *holo-*BpsA in 50 mM Tris-Cl buffer pH 7.8, 40% (v/v) glycerol was incubated with PcpS and Coenzyme A to convert any remaining *apo*-BpsA to the *holo-*form. In a 96 well plate four distinct reaction mixes were established in triplicate, containing either 2 μM *apo*-BpsA, 2 μM *holo*-BpsA, 2 μM *holo*-BpsA and 0.25 μM PcpS, or 0.25 μM PcpS alone, in 10 mM MgCl_2,_ 50 mM Tris-Cl pH 7.8, 40 μM Coenzyme A and ddH_2_O to a final volume of 50 μL. The reaction mixes were incubated for 30 min at 200 rev/min, 30 °C. Indigoidine synthesis was then tested as previously, and the initial velocity of the reaction was calculated by measuring the maximum slope value (as previously described^26^).

### Kinetic analysis of *holo*-BpsA

To confirm that BpsA had been converted to the active *holo* form, the rate of indigoidine synthesis was measured. A reaction mix containing ddH_2_O, 50 mM Tris-Cl pH 8.5, 20 mM MgCl_2_, 6 mM ATP and 1 μM BpsA in a total volume of 90 μL was added to individual wells of a 96 well plate in triplicate. The reaction was initiated by the addition of 10 μL of 1 mM L-glutamine in ddH_2_O, giving a final reaction volume of 100 μL per well. The 96 well plate was mixed at 1000 rev/min for 15 s and A_590_ values were recorded every 10 s for 1 h. The reaction rate was calculated by measuring the maximum reaction velocity (∆A_590_ s^−1^).

### Kinetic analysis of L-glutamine samples

To estimate the concentration of glutamine samples without resolubilising the indigoidine produced, the maximum absorbance achieved across a range of concentrations was measured. A reaction mix containing: ddH_2_O, 50 mM Tris-Cl pH 8.5, 20 mM MgCl_2_, 6 mM ATP and 2 μM *holo-*BpsA in a total volume of 90 μL was added to a 96 well plate in triplicate. The reaction was initiated by the rapid addition of 10 μL of L-glutamine standards spanning a range of concentrations (0–1000 μM). The 96 well plate was mixed at 1000 rev/min for 15 s and A_590_ values were measured every 10 s for 1 h. The maximum A_590_ value achieved for each sample within this time was recorded and this was used to generate a standard curve.

### Optimisation of DMSO stop method

The DMSO stop protocol comprises two parts. In the first part *holo*-BpsA converts two molecules of glutamine into indigoidine. A reaction mix containing the following was set up: 50 mM Tris-Cl pH 8.5, 20 mM MgCl_2_, 6 mM ATP and 3 μM *holo*-BpsA in ddH_2_O. 30 μL of reaction mix was added in triplicate to a 96 well plate containing 10 μL of L-glutamine standards spanning a range of concentrations (0–1000 μM) in ddH_2_O to initiate the reaction. The 96 well plate was then incubated (25 °C 200 rev/min for 60 min). Once all the L-glutamine had been converted to indigoidine it was re-solubilised by the addition of 200 μL anhydrous DMSO (final concentration 83% (v/v)). The 96 well plate was then shaken at 2,000 rev/min for 20 min and the A_590_ value for each well was recorded. Triplicate reactions were averaged and used to generate a standard curve. For each of the different optimisation steps individual parameters were varied including incubation time, DMSO concentration and BpsA concentration, to ensure complete conversion of L-glutamine to indigoidine and complete re-solubilisation of indigoidine.

### Conversion of indigoidine to *leuco* indigoidine

A standard curve with a range from 0–1000 μM was set up in triplicate as described in the preceding section. The protocol of Müller *et al*.^22^ was then used to covert the indigoidine to *leuco* indigoidine. For this, 2.3 μL of a solution of 0.15 g of sodium dithionate in 10 ml of 1 M NaOH was added to each well. The 96 well plate was then shaken at 2,000 rev/min for 2 min, during which time a complete loss of colour was observed. The relative fluorescence was then recorded in a platereader using a 415 nm excitation wavelength and 520 nm emission wavelength, and these data used to generate a standard curve.

### Measurement of glutamine in a range of relevant conditions

For LB, DMEM and MCDB no additional sample processing was needed. For blood (collected using a purple Vacutainer blood collection tube) and urine the samples were centrifuged (13,000 *g*, 2 min, 25 °C) to remove cells. The blood sample also required additional processing. The plasma was passed through a 3000 Dalton cut off column via centrifugation (2,700 *g,* 30 min, 25 °C) to deproteinate the sample. L-glutamine standards (0–1000 μM) were prepared in ddH_2_O. 30 μL of reaction mix (prepared as for the “Optimisation of DMSO stop method”, above) was added to 10 μL of sample to initiate the reaction. The 96 well plate was then incubated (25 °C, 200 rev/min, 60 min). The indigoidine was then re-solubilised by the addition of 200 μL of DMSO. The 96 well was mixed (2,000 rev/min, 20 min) and the A_590_ value was recorded. Each reaction was repeated in triplicate and the A_590_ values averaged to generate the final value. A standard curve was generated from the standards and this was used to extrapolate the calculated glutamine concentrations for the different samples.

### Long term stability

The long term stability tests at different temperatures were performed on *apo*-BpsA which was converted to *holo*-BpsA immediately prior to testing. Three samples were stored for 24 weeks in storage buffer (40% (v/v) glycerol, 50 mM sodium phosphate buffer pH 7.8) at −20 °C, 4 °C or 25 °C. A reaction mix containing the following reagents was generated to convert the *apo*-BpsA to *holo*-BpsA: 2 μM *apo-*BpsA, 12.5 μM CoA, 0.1 μM Sfp, 5 mM MgCl_2_, 50 mM Tris-Cl pH 7.8 and ddH_2_O. A total volume of 25 μL per reaction was incubated at 30 °C 200 rev/min for 30 min. 25 μL of this reaction mix was dispensed into a 96 well plate containing 50 μL of 50 mM Tris-Cl pH 7.8 and 5 mM MgCl_2_ in ddH_2_O. Indigoidine synthesis was initiated by the addition of 25 μL of 5 mM ATP and 2 mM L-glutamine in ddH_2_O (concentrations are per final 100 μL reaction volume). The 96 well plate was then shaken at 1000 rev/min for 10 s and the A_590_ values were recorded every 20 s for 1 h. The initial velocity of the reaction was calculated by finding the maximum slope value (as previously described^26^).

The 11 month stability test was performed using the same preparation of *holo-*BpsA. A master mix containing the following was dispensed into a 96 well plate 2 μM *holo*-BpsA, 50 mM Tris-Cl pH 8.5, 20 mM MgCl_2_ and 5 mM ATP and ddH_2_O with a final volume of 90 μL. The reaction was initiated by the addition of 10 μL of 1000 μM L-glutamine in ddH_2_O. The 96 well plate was then shaken at 1000 rev/min for 10 s and the A_590_ values were recorded every 10 s for 1 h. The velocity of the reaction was calculated by finding the maximum slope value as above. The *holo*-BpsA was stored for 11 months at −20 °C in storage buffer. The sample was then tested in the same manner and the maximum velocity was calculated.

## Additional Information

**How to cite this article**: Brown, A. S. *et al*. A sensitive single-enzyme assay system using the non-ribosomal peptide synthetase BpsA for measurement of L-glutamine in biological samples. *Sci. Rep.* **7**, 41745; doi: 10.1038/srep41745 (2017).

**Publisher's note:** Springer Nature remains neutral with regard to jurisdictional claims in published maps and institutional affiliations.

## Supplementary Material

Supplementary Material

## Figures and Tables

**Table 1:** Measurement of L-glutamine in spiked biological samples.

Sample	Calculated value (μM)	A_590_ value
LB + 400 μM L-glutamine	382.7 ± 6.7	0.164 ± 0.002
DMEM + 400 μM L-glutamine	410.9 ± 14.1	0.173 ± 0.006
MCDB + 400 μM L-glutamine	411.6 ± 12.5	0.173 ± 0.001
H_2_O + 400 μM L-glutamine	420.4 ± 7.3	0.181 ± 0.003
Blood	240.1 ± 6.0	0.117 ± 0.002
Blood + 400 μM L-glutamine	610.3 ± 20.3	0.220 ± 0.006
Urine	248.4 ± 2.1	0.119 ± 0.001
Urine + 400 μM L-glutamine	631.7 ± 11.4	0.226 ± 0.003
H_2_O	−0.4 ± 13.7	0.049 ± 0.004
H_2_O + 400 μM L-glutamine	396.0 ± 19.1	0.160 ± 0.005
