# Supplementary material for: A sensitive single-enzyme assay system using the non-ribosomal peptide synthetase BpsA for measurement of L-glutamine in biological samples
Source: Sci Rep. 2017 Jan 31;7:41745. doi: 10.1038/srep41745 (PMC5282505; doi:10.1038/srep41745)
Supplement: Supplementary Material [file srep41745-s1.doc]

**Supplementary Material**

**A sensitive single-enzyme assay system using the non-ribosomal peptide synthetase BpsA for measurement of L-glutamine in biological samples**

Alistair S Brown^1^, Katherine J Robins^1^ and David F Ackerley*^1,2^

^1^School of Biological Sciences, Victoria University of Wellington, Wellington, New Zealand

^2^Centre for Biodiscovery, Victoria University of Wellington, Wellington, New Zealand

*Correspondence to [david.ackerley@vuw.ac.nz](mailto:david.ackerley@vuw.ac.nz)

*Table of contents*

**Figure S1: Derivation of a standard curve from the maximal initial rates of indigoidine synthesis**

**Figure S2: Converting indigoidine to its colourless *leuco* form does not provide a more accurate means of measuring L-glutamine**

**Figure S3: Raising the concentration of *holo*-BpsA from 3 µM to 15 µM increases the reaction velocity**


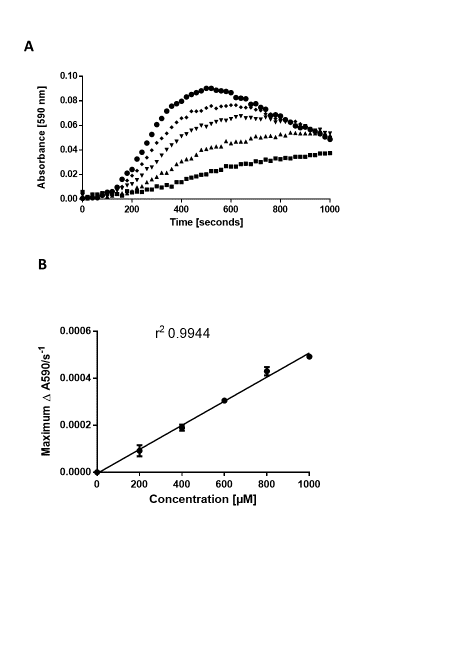


**Figure S1. Derivation of a standard curve from the maximal initial rates of indigoidine synthesis**. **A**. A master mix containing 2 µM holo-BpsA, 50 mM Tris-Cl pH 8.5, 20 mM MgCl_2_, 5 mM ATP and ddH_2_O to a final volume of 90 µl was added to individual wells of a 96 well plate. To initiate the reaction, 10 µL of L-glutamine stock solutions were added to the following concentrations: 1,000 µM (●), 800 µM (♦), 600 µM (▼), 400 µM (▲), or 200 µM (■) L-glutamine. A_590_ values were recorded for each well every 20 s. Each data point is the average of three technical replicates, normalised against the mean value for 0 µM L-glutamine (not shown on graph). **B.** The maximal velocity of each reaction depicted in panel A was calculated by finding the maximum slope value [as previously described by Owen, J. G., Copp, J. N. & Ackerley, D. F. Rapid and flexible biochemical assays for evaluating 4′-phosphopantetheinyl transferase activity. *Biochemical Journal* 436, 709–717 (2011)]. Data are the mean values of three replicates normalised against the 0 µM L-glutamine standard, and error bars indicate standard error of the mean.


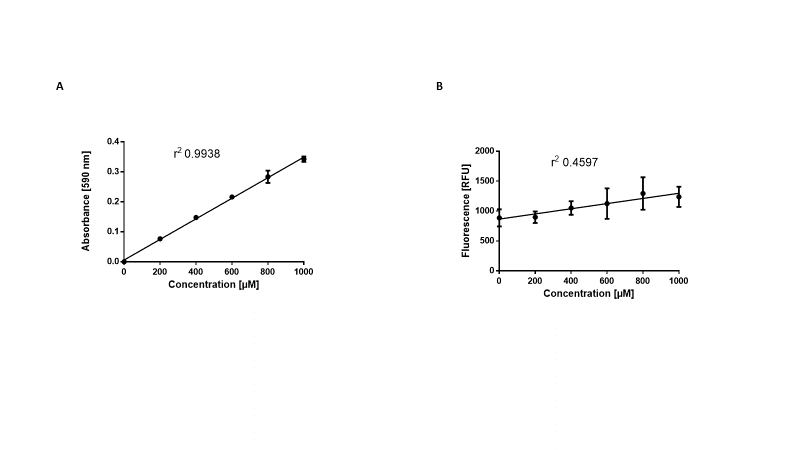


**Figure S2. Converting indigoidine to its colourless *leuco* form does not provide a more accurate means of measuring L-glutamine**. **A**. A linear standard curve was generated by incubating 10 µL of L-glutamine standards across a range of concentrations (0 -1000 µM) with 30 µL of a reaction mix (50 mM Tris-Cl pH 8.5, 10 mM MgCl_2_, 5 mM ATP, 3 µM holo-BpsA in ddH_2_O) for 1 h at 25 °C. This was followed by resolubilisation in 200 µL DMSO and a further incubation with shaking at 2,000 rev/min for 20 min at 25 °C. Data are the means of three replicates and error bars indicate standard error of the mean. **B.** After the standard curve was recorded, 2.3 µL of a reducing agent (0.15g of sodium dithionate in 10 ml of 1 M NaOH) was added to each well to convert all indigoidine into the colourless *leuco* form. Following 2 min incubation at 2,000 rev/min, fluorescence measurements (ex 415 nm / em 520 nm) were used to generate a standard curve. The low r^2^ value indicates that this is not a reliable means of measuring L-glutamine.

**Figure S3.** **Raising the concentration of *holo*-BpsA from 3 µM to 15 µM increases the reaction velocity.** Two 30 µL reaction mixes comprising 50 mM Tris-Cl pH 8.5, 10 mM MgCl_2_, 6 mM ATP and either 3 µM (black bars) or 15 µM (grey bars) *holo-*BpsA were established in a 96 well plate. The reaction was initiated by the addition of 10 µL of 1000 µM L-glutamine. Replicate reactions were halted at 5 min intervals by the addition of 200 µL DMSO. After 15 min the entire plate was shaken at 2,000 rev/min for 20 min to resolubilise the indigoidine, and A_590_ values were recorded for each timepoint. Data are the means of three replicates and error bars indicate standard error of the mean.
